# Supplementary material for: Integrative Study of the Life Cycle in the Marine Protist Thraustochytrium aureum ssp. strugatskii
Source: Int J Mol Sci. 2025 Nov 22;26(23):11302. doi: 10.3390/ijms262311302 (PMC12692134; doi:10.3390/ijms262311302)
Supplement: Supplementary file 1 [file ijms-26-11302-s001.zip › Supplementary Figures.pdf]

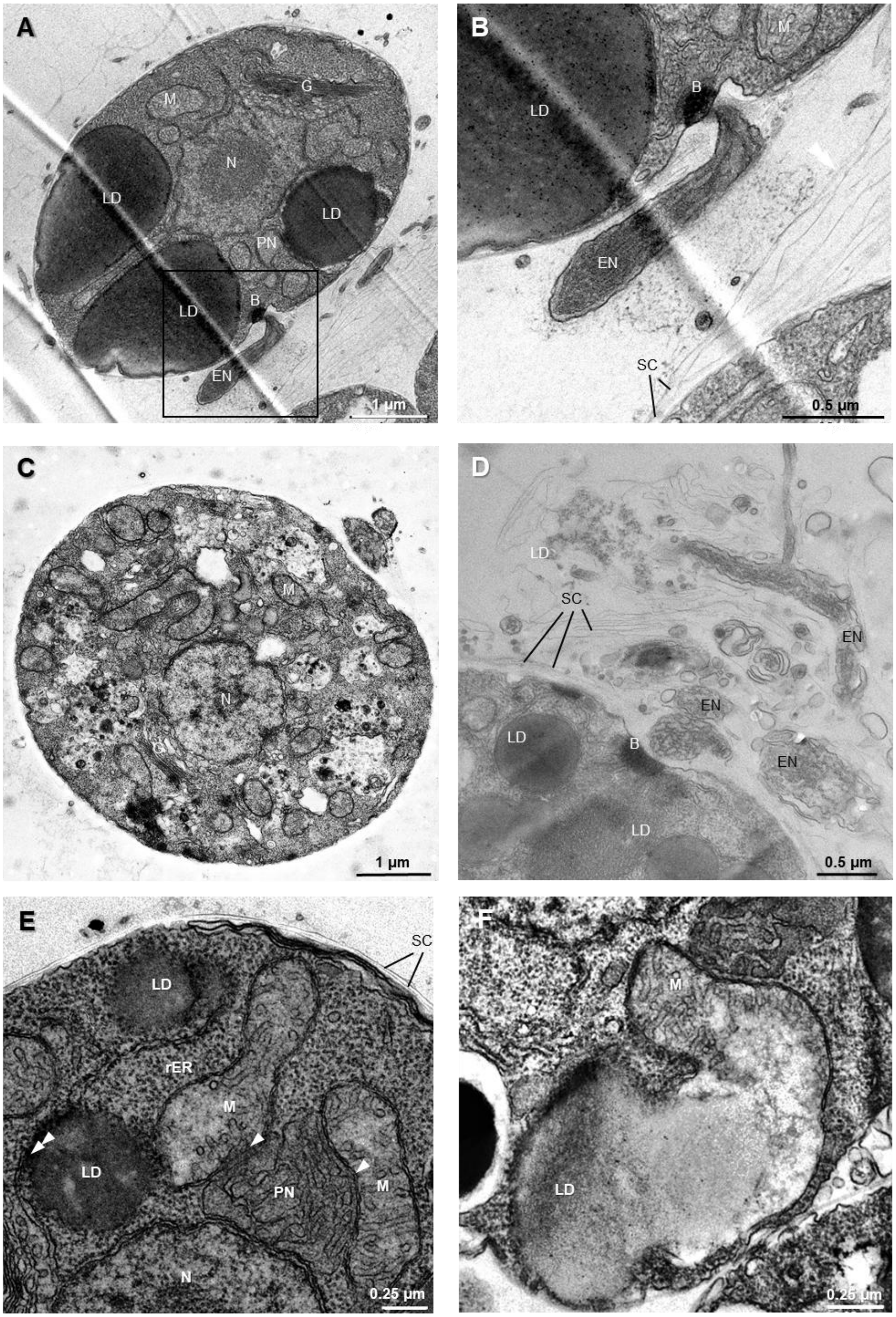

Figure S1. Morphology of the vegetative cell of *Thraustochytrium aureum* ssp. *strugatskii*. **(A)** TEM image of a vegetative cell containing large lipid droplets (LD) in the cytoplasm and an ectoplasmic net (EN) emerging through the bothrosome (B). **(B)** TEM image showing the bothrosome (B) and an ectoplasmic reticulum thread of the same cell at high magnification. **(C)** TEM image of a vegetative cell lacking large lipid droplets. **(D)** TEM image showing the bothrosome (B) in a vegetative cell and a cross section of endoplasmic reticulum filaments revealing internal membrane structures. **(E-F)** Organellar contacts within vegetative cells of *T. aureum* ssp. *strugatskii*. **(E)** Examples of contact sites between different organelles. **(F)** Fusion of mitochondria (M) with lipid droplets (LD).

Abbreviations: EN, ectoplasmic net; N, nucleus; M, mitochondrion; G, Golgi apparatus; PN, paranuclear body; LD, lipid droplet; B, bothrosome; SC, scales; rER, rough endoplasmic reticulum. Single arrowhead, contact between the paranuclear body and mitochondria; double arrowhead, contact between the endoplasmic reticulum and lipid droplet.

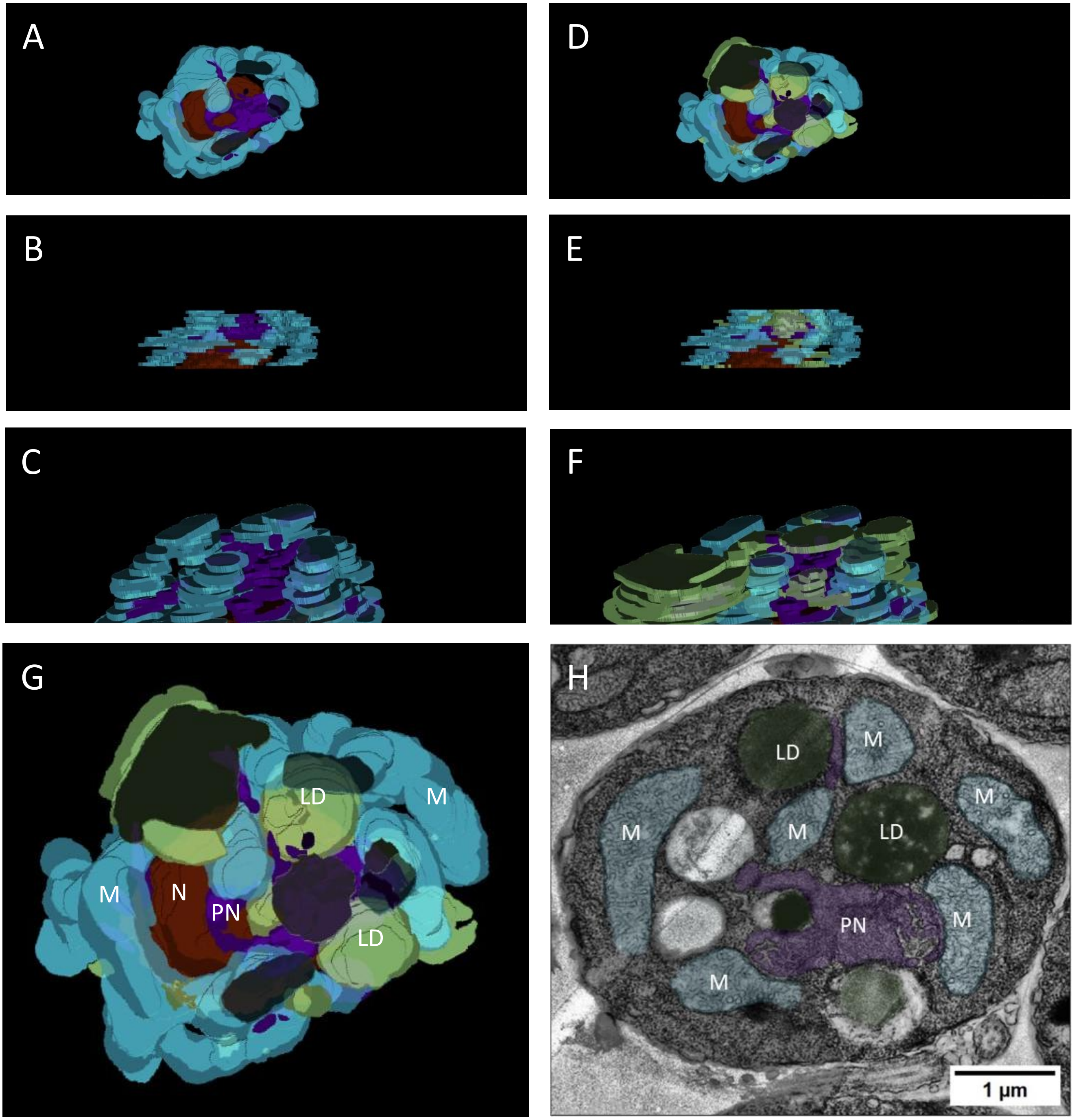

Figure S2.
Three-dimensional reconstruction and ultrastructure of undispersed zoospores of *Thraustochytrium aureum* ssp. *strugatskii*.
(A) Top view of the 3D model (lipid inclusions omitted);
(B) Side view (with lipid granules);
(C) Oblique top view (with lipid granules);
(D) Top view (with lipid inclusions);
(E) Side view (with lipid inclusions);
(F) Oblique top view (with lipid inclusions);
(G) Complete 3D rendering of undispersed zoospores ;
(H) TEM image of undispersed zoospores showing the nucleus (N), mitochondria (M), paranuclear body (PN), and large lipid droplets (LD).
Abbreviations: N, nucleus; M, mitochondrion; G, Golgi apparatus; PN, paranuclear body; LD, lipid droplet.

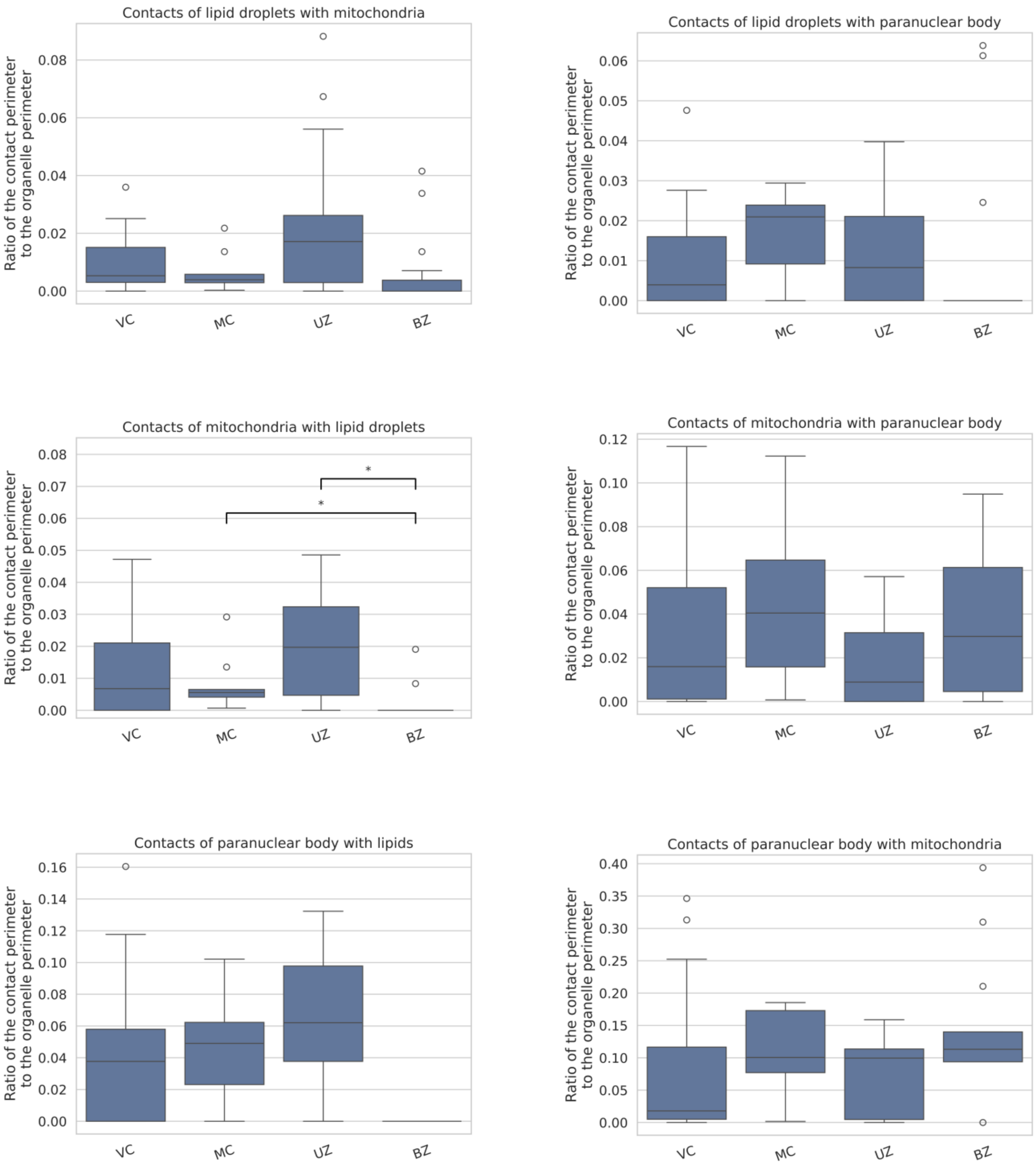

Figure S3. Quantitative analysis of organelle contacts in *Thraustochytrium aureum* ssp. *strugatskii* at different life-cycle stages.

Data are presented as boxplots (median ± 1.5 IQR).

Significance levels: *$p < 0.05$, **$p < 0.01$, ***$p < 0.001$.

Abbreviations: VC, vegetative cell (juvenile sporangium); MC, multinucleated cell (mature sporangium); UZ, undispersed zoospore; BZ, biflagellate zoospore.

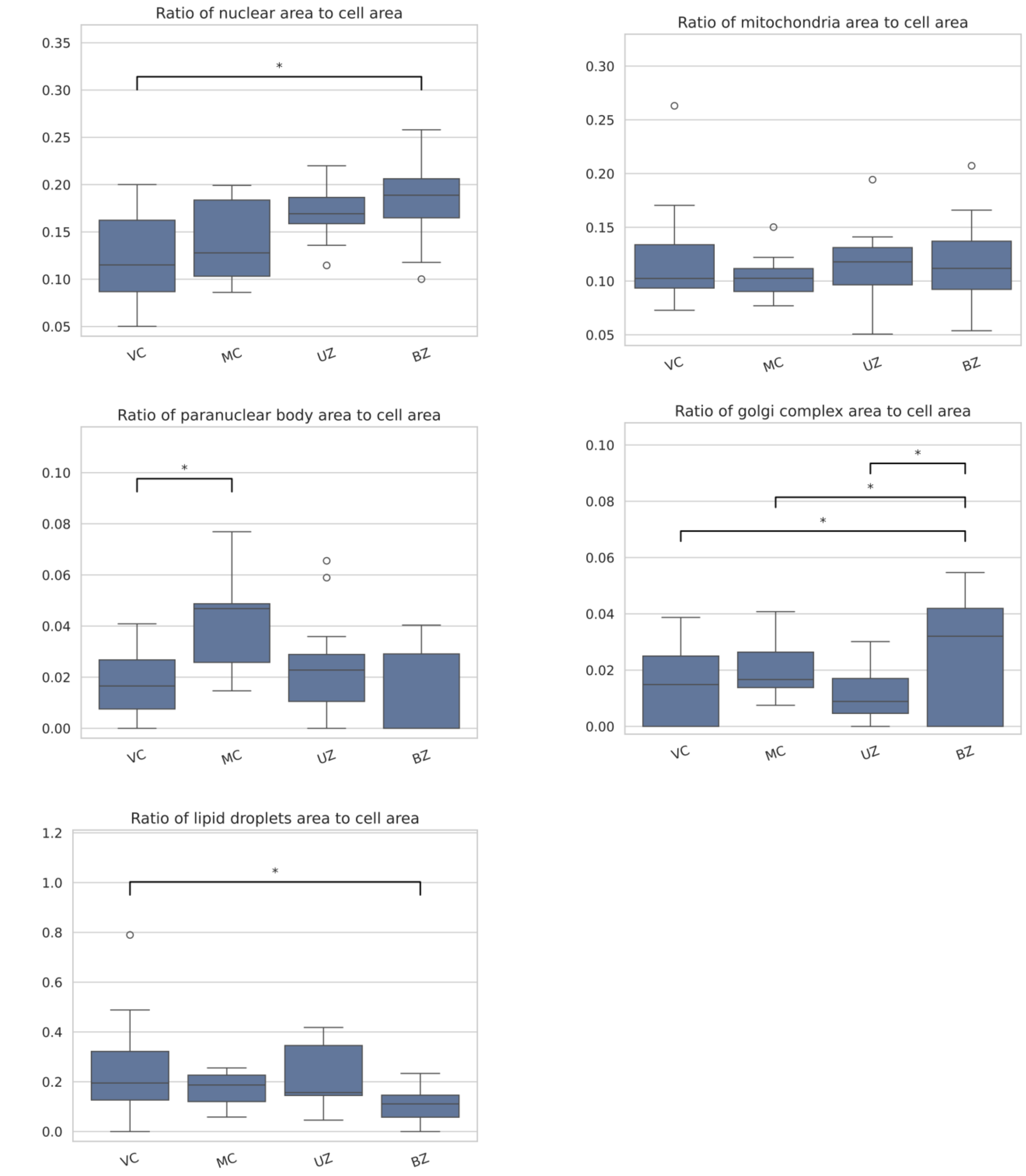

Figure S4. Comparison of relative organelle areas in *Thraustochytrium aureum* ssp. *strugatskii* at different life-cycle stages.

Data are presented as boxplots (median ± 1.5 IQR).

Significance levels: *$p < 0.05$, **$p < 0.01$, ***$p < 0.001$.

Abbreviations: VC, vegetative cell (juvenile sporangium); MC, multinucleated cell (mature sporangium); UZ, undispersed zoospore; BZ, biflagellate zoospore.

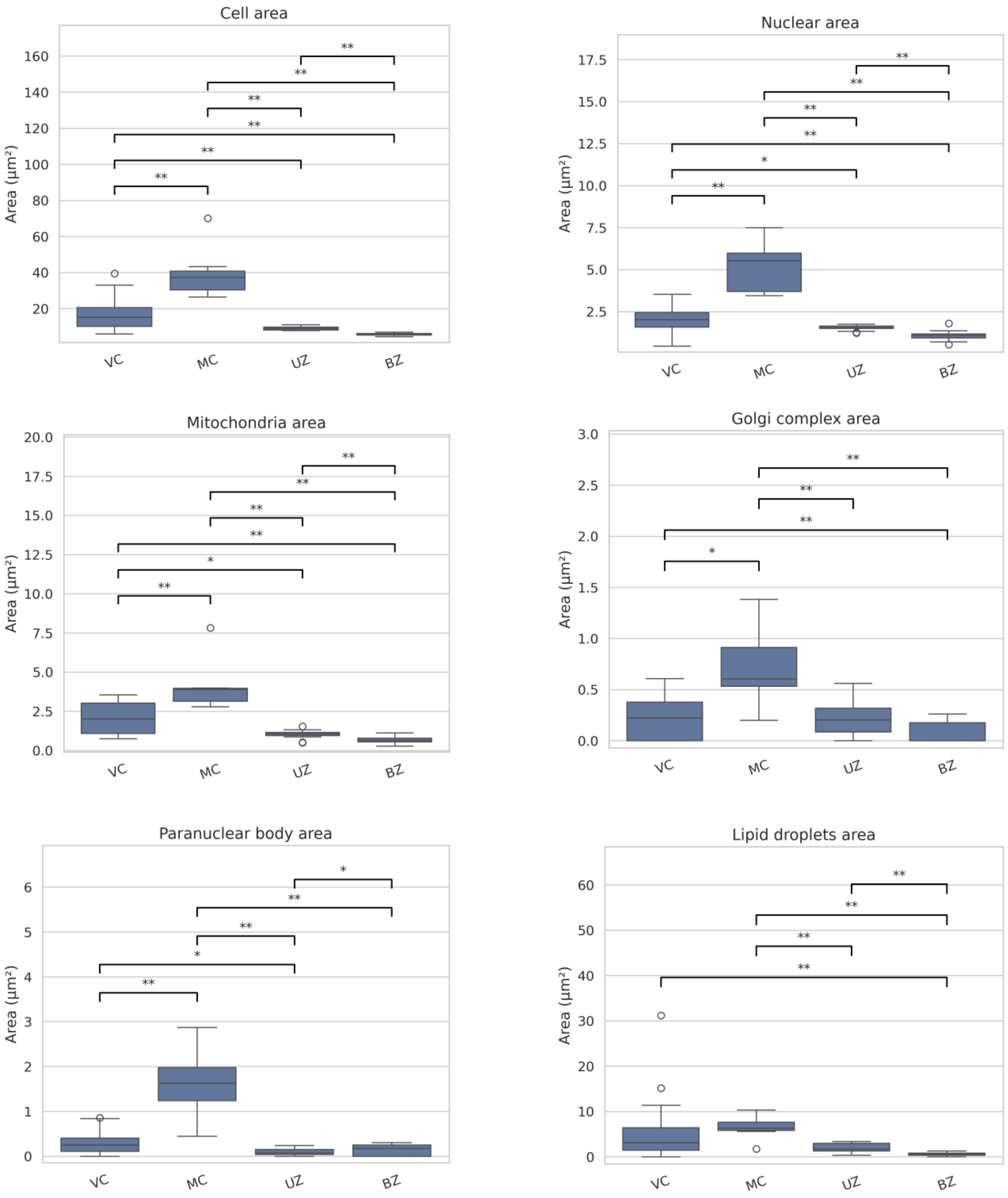

Figure S5. Comparison of organelle areas in *Thraustochytrium aureum* ssp. *strugatskii* at different life-cycle stages.
Data are presented as boxplots (median ± 1.5 IQR).
Significance levels: *$p < 0.05$, **$p < 0.01$, ***$p < 0.001$.
Abbreviations: VC, vegetative cell (juvenile sporangium); MC, multinucleated cell (mature sporangium); UZ, undispersed zoospore; BZ, biflagellate zoospore.

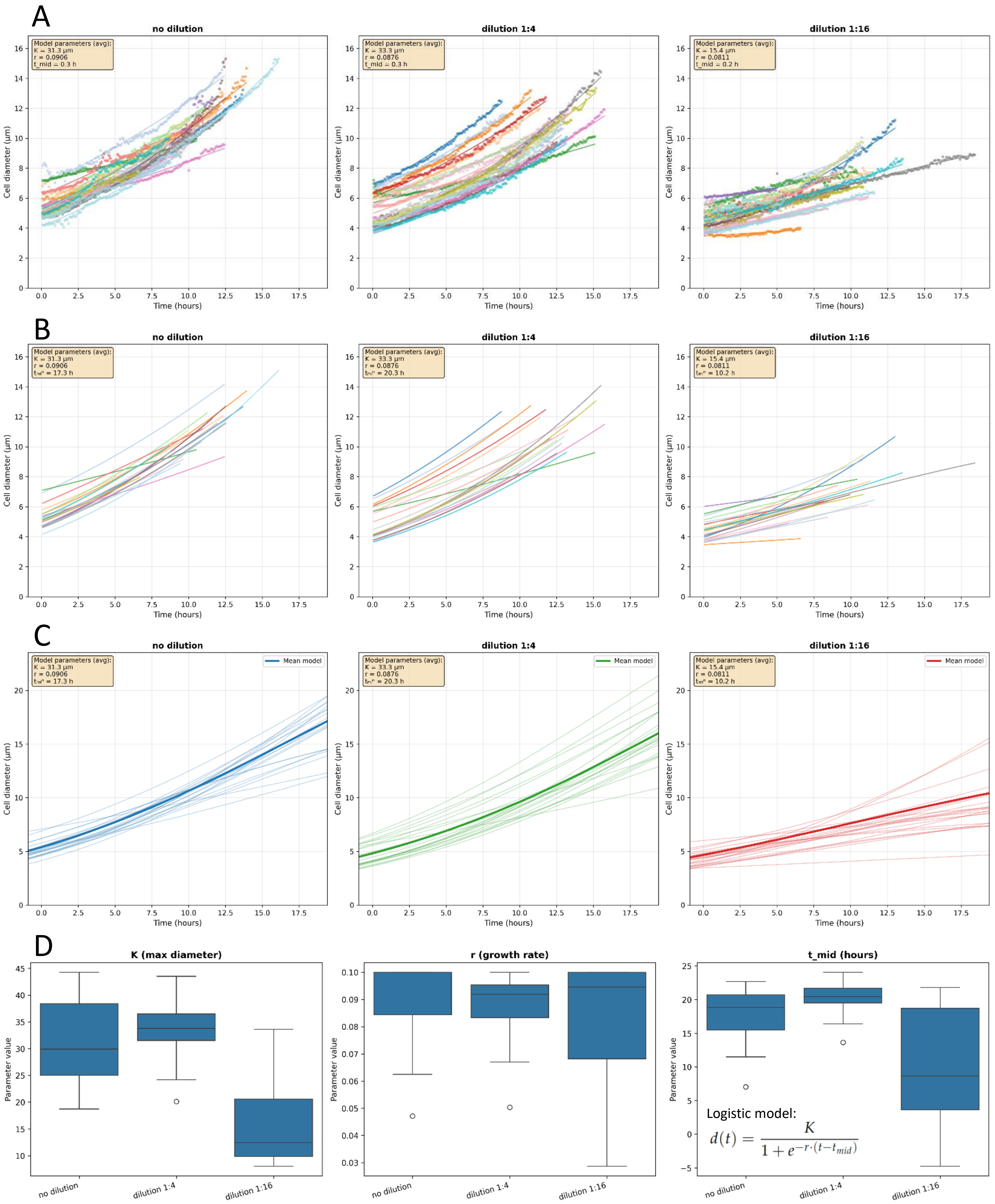

Figure S6. Logistic modeling of *Thraustochytrium aureum* ssp. *strugatskii* cell growth dynamics under three dilution conditions of FAND medium.

(A) Experimental data (see Table S3) and fitted logistic growth models for all conditions. **(B)** Individual logistic growth curves for each dilution. **(C)** Model-based predictions of cell growth dynamics over time. Each panel shows average OD dynamics for three dilution levels: undiluted (blue), 1:4 dilution (orange), and 1:8 dilution (green). **(D)** Estimated model parameters: ***K*** represents the maximum cell diameter (growth capacity), ***r*** denotes the growth rate parameter, and ***t_mid*** indicates the time required to reach half of the maximum size.

Logistic models accurately describe cell growth under all tested nutrient concentrations and were further used to estimate division rates and growth kinetics.
